# Volume Estimation of the Thalamus Using Freesurfer and Stereology: Consistency between Methods

**DOI:** 10.1007/s12021-012-9147-0

**Published:** 2012-04-06

**Authors:** Simon S. Keller, Jan S. Gerdes, Siawoosh Mohammadi, Christoph Kellinghaus, Harald Kugel, Katja Deppe, E. Bernd Ringelstein, Stefan Evers, Wolfram Schwindt, Michael Deppe

**Affiliations:** 1 Department of Neurology, University of Münster, Münster, Germany; 2 Department of Clinical Neuroscience, Institute of Psychiatry, King’s College London, London, UK; 3 Wellcome Trust Centre for Neuroimaging, UCL Institute of Neurology, University College London, London, UK; 4 Department of Neurology, Klinikum Osnabrück, Osnabrück, Germany; 5 Department of Clinical Radiology, University of Münster, Münster, Germany; 6 Department of Radiology, Klinikum Osnabrück, Osnabrück, Germany; 7 Universität Münster, Klinik und Poliklinik für Neurologie, Funktionelle Bildgebung, Albert-Schweitzer-Campus 1, Gebäude A1, 48129 Münster, Germany

**Keywords:** FreeSurfer, Diffusion tensor imaging, Fractional anisotropy, MR image analysis, Stereology, Volume

## Abstract

Freely available automated MR image analysis techniques are being increasingly used to investigate neuroanatomical abnormalities in patients with neurological disorders. It is important to assess the specificity and validity of automated measurements of structure volumes with respect to reliable manual methods that rely on human anatomical expertise. The thalamus is widely investigated in many neurological and neuropsychiatric disorders using MRI, but thalamic volumes are notoriously difficult to quantify given the poor between-tissue contrast at the thalamic gray-white matter interface. In the present study we investigated the reliability of automatically determined thalamic volume measurements obtained using FreeSurfer software with respect to a manual stereological technique on 3D T1-weighted MR images obtained from a 3 T MR system. Further to demonstrating impressive consistency between stereological and FreeSurfer volume estimates of the thalamus in healthy subjects and neurological patients, we demonstrate that the extent of agreeability between stereology and FreeSurfer is equal to the agreeability between two human anatomists estimating thalamic volume using stereological methods. Using patients with juvenile myoclonic epilepsy as a model for thalamic atrophy, we also show that both automated and manual methods provide very similar ratios of thalamic volume loss in patients. This work promotes the use of FreeSurfer for reliable estimation of global volume in healthy and diseased thalami.

## Introduction

The thalamus is of central interest in many disorders of the nervous system (Andreasen 1997; Dom, et al. 1976; Lee and Marsden 1994; Meador-Woodruff, et al. 2003; Speedie and Heilman 1983; Williams 1965; Xuereb, et al. 1991). The functioning of the thalamus is crucial to many sensory, motor and cognitive systems, and therefore has also been subject to a great deal of investigation in the cognitive neurosciences (Basso, et al. 2005; Engelborghs, et al. 1998; Herrero, et al. 2002). It is in these capacities that analysis of thalamic structure and function is a continually researched theme in neuroimaging investigations, particularly using magnetic resonance imaging (MRI). Analysis of volume or shape using MRI techniques may provide important information with respect to the involvement of the thalamus in neurological and neuropsychiatric disorders, including generalized (Du, et al. 2011; Pulsipher, et al. 2009) and partial (Gong, et al. 2008; Pulsipher, et al. 2007) epilepsy, schizophrenia (Adriano, et al. 2010), Huntington’s disease (Douaud, et al. 2006; Kassubek, et al. 2005), Parkinson’s disease (McKeown, et al. 2008), and Alzheimer’s disease (de Jong, et al. 2008). Reliable measurement of thalamic structure is, however, notoriously difficult to achieve, particularly given the typically poor between-tissue MR contrast of the thalamic nuclei and adjacent white matter (Amini, et al. 2004). It is therefore important to develop new and improve and validate existing methodologies that provide thalamic metrics. Like for other subcortical brain structures, there are several approaches freely available to estimate thalamic volume from MR images. At either end of the MR image analysis spectrum, there are manual and automated approaches; manual approaches are user-dependent, time consuming but are considered to be the gold standard of MR image analysis techniques (Bonilha, et al. 2004; Collins and Pruessner 2010; Crum, et al. 2001; Pruessner, et al. 2000). Automated approaches remove the need of an expert anatomist, are dependent on computer algorithms, and are time efficient, but require a great deal of validation against manual methods to determine the specificity and validity of measurements (Chupin, et al. 2009; Morra, et al. 2008). The primary goal of the present study was to evaluate the validity of thalamic volume measurements obtained from a frequently used automated approach with respect to a reputable manual approach widely used in the imaging, anatomical and histological sciences.

The fully automated approach investigated in the present study was the subcortical segmentation and volume estimation techniques (Fischl, et al. 2002) incorporated into FreeSurfer software (http://surfer.nmr.mgh.harvard.edu/), which provide observer-independent volumes for individual subcortical nuclei from conventional MR images. Similarly to other methods that automatically segment and estimate subcortical volume such as FIRST (Patenaude, et al. 2011) incorporated into FSL software (http://www.fmrib.ox.ac.uk/fsl/first/index.html), there has been a recent proliferation of studies using FreeSurfer methods for volumetric studies, some of which have included comparison with manual methods, most notably for the hippocampus (Cherbuin, et al. 2009; Dewey, et al. 2010; Morey, et al. 2009; Pardoe, et al. 2009; Shen, et al. 2010; Tae, et al. 2008). To our knowledge, there has been no independent comparison between manual methods and FreeSurfer methods for volume estimation of the thalamus. The manual approach used to evaluated FreeSurfer-based thalamic volumes in the present study was the Cavalieri method of design-based stereology in conjunction with point counting (Gundersen and Jensen 1987; Gundersen, et al. 1999; Mayhew 1992; Roberts, et al. 2000), which is a 100 % investigator interactive technique that requires manual determination of sampling density for a given brain structure (i.e. the stereological parameters necessary to produce a reliable volume estimate) and investigator decisions on whether or not sampling probes (i.e. points) intersect the brain region-of-interest (ROI). Stereology requires the use of a human anatomist with expert knowledge of anatomical boundaries that divide legitimate (i.e. thalamic) and illegitimate (i.e. non-thalamic) brain tissue. Manual approaches such as stereology are considered gold standard because it is assumed that human knowledge and perception is superior to computer algorithms that determine regional brain boundaries.

We examined the consistency between manual and automated thalamic volume estimation in two ways. Firstly, we compared thalamic volume estimates in a sample of neurologically and psychiatrically healthy subjects. Secondly, we compared the methods in their sensitivity in detecting thalamic atrophy in patients with juvenile myoclonic epilepsy (JME). JME is an electro-clinical syndrome that by definition is non-lesional and without abnormality on conventional magnetic resonance imaging (MRI) (Berg, et al. 2010; ILAE 1989), but has previously been shown to be associated with thalamic structural alterations (Deppe, et al. 2008; Kim, et al. 2007; Mory, et al. 2011; Pulsipher, et al. 2009), and is generally considered to be intimately associated with thalamic dysfunction (Holmes, et al. 2010). We therefore compared morphometric approaches for volume estimation of both healthy and diseased thalami.

## Methods

### Participants

We studied a neurologically and psychiatrically healthy control group that was composed of 62 adult volunteers (32 females, mean age 27.9 ± 4.3 SD, range 21–43), all of whom had normal neurological examination and normal MRI (T1-, T2-weighted, and FLAIR). We also studied ten patients (6 females, mean age 28.6 ± 8.8 SD, range 19–42) with JME. Clinical information for these patients can be found elsewhere (Deppe, et al. 2008; Keller, et al. 2011a). There was no statistical difference in age between patients and controls (*t* = 0.38, *p* = 0.70). All subjects gave written informed consent and the local ethics committee approved this study.

### Magnetic Resonance Imaging

All participants underwent high resolution MRI T1-weighted, T2-weighted and FLAIR imaging at 3 T (Philips Intera T30, T/R head coil). All MRI modalities were used to exclude the possibility of brain lesions in patients and controls. For volumetric analysis, we acquired T1-weighted structural MRIs using a high-resolution 3D turbo-field-echo sequence (matrix 256 × 256 × 160 over a field of view of 25.6 × 25.6 × 16 cm^3^ reconstructed after zero filling to 512 × 512 × 320 cubic voxels with an edge length of 0.5 mm). Prior to morphometric analyses, all MR images were intensity inhomogeneity corrected and resampled to isotropic voxels of 1 × 1 × 1 mm (256 × 256 × 160 slices) using in-house software (Eval 3.0). To confirm that there was no systematic difference in head size between patient and control groups, we automatically obtained relative brain size, VSCALE (global tissue volumes normalized for head size) and CSF volume estimates from the 3D T1-weighted images of all subjects using the SIENAX protocol (Smith, et al. 2002) integrated into FSL software (http://www.fmrib.ox.ac.uk/fsl/siena/index.html). There were no inter-group differences in relative brain size (*p* = 0.40), VSCALE (*p* = 0.96) or CSF volume (*p* = 0.95).

A. Stereology

The Cavalieri method of design-based stereology in conjunction with point counting (Gundersen and Jensen 1987; Gundersen, et al. 1999; Mayhew 1992; Roberts, et al. 2000) was used as an unbiased estimator of the volume of the left and right thalamus in all subjects. By using the Cavalieri method, volume is directly estimated from equidistant and parallel MR images of the brain with a uniform random starting position. A second level of sampling is required to estimate the section area from each image by applying point counting within the ROI. The mathematical justification and implementation of the methodology is simple and it can be applied to structures of arbitrary shape (Garcia-Finana, et al. 2009). This technique has been frequently applied to reliably estimate brain volume and surface area on MR images (Acer, et al. 2010; Bas, et al. 2009; Cowell, et al. 2007; Eriksen, et al. 2010; Hallahan, et al. 2011; Howard, et al. 2003; Jelsing, et al. 2005; Keller, et al. 2009; Keller, et al. 2007; Keller, et al. 2002a; Keller, et al. 2009b; Keller, et al. 2002b; Lux, et al. 2008; Mackay, et al. 1998; Mackay, et al. 2000; Ronan, et al. 2006; Salmenpera, et al. 2005; Sheline, et al. 1996), and more widely applied to study other aspects of anatomy with and without the use of MRI. Stereology has been shown to be at least as precise as tracing and thresholding volumetry techniques and substantially more time efficient, with validation relative to post-mortem measurements (Garcia-Finana, et al. 2003; Garcia-Finana, et al. 2009; Keller and Roberts 2009; Keshavan, et al. 1995). Windows-compatible Easymeasure software (Keller, et al. 2007; Puddephat 1999) was used for point counting on MR images.

**Fig. 1:**
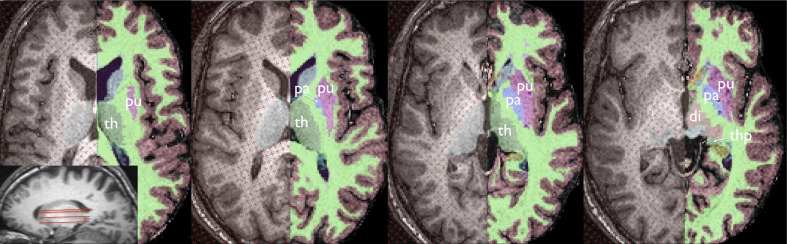
Stereological and FreeSurfer methods used to estimate thalamic volume shown at approximately the same axial sections. Both methods are shown for the same hemisphere (the FreeSurfer axial sections are flipped to show maximal correspondence between techniques) of the same randomly selected subject. The sagittal MR section illustrates the approximate levels of the axial sections shown. Abbreviations: di, ventral diencephalon (*peach*); pa, pallidum (*violet*); put, putamen (*lilac*); th, thalamus (*dark green*); thp, pulvinar of the thalamus (*dark green*)

Given that the borders of different thalamic nuclei are almost indistinguishable on conventional MRI, the thalamus was sampled as an entire complex, including the anterior thalamic nucleus, mediodorsal thalamic nucleus, lateral dorsal thalamic nucleus, ventral lateral thalamic nucleus, ventral postero-lateral/medial thalamic nuclei, and pulvinar (Duvernoy 1999). The lateral and medial geniculate nuclei were not included in measurements, similar to previous studies (Natsume, et al. 2003; Qiu, et al. 2009). On axial sections, point counting began on the dorsal most section, where the area of the lateral dorsal thalamic nucleus was demarcated laterally by the corona radiata and medially by the lateral ventricles. As measurements progressed ventrally, the posterior limb of the internal capsule bordered the thalamus laterally. On ventral sections, care was taken to delineate the thalamic nuclei from the neighbouring pars medialis of the globus pallidus, and at the most ventral sections, to segregate the pulvinar and area of the ventral posterolateral nucleus from the emerging hypothalamus anteriorly, superior colliculus posteromedially, and the hippocampus posteriorly / posterolaterally. Figure 1 shows point counting within the thalamic ROI relative to the automated FreeSurfer approach described below. More information on the MRI-based anatomy of the thalamus with respect to post-mortem sections can be found at http://www.psychiatry.uiowa.edu/mhcrc/pdf/papers/thalamus.pdf.

The sampling density (i.e. size of points, distance between sections) were optimized to achieve a coefficient of error (CE) of less that 5 % (Roberts, et al. 2000), an approach that has been adopted in our stereological analysis of the hippocampus (Keller, et al. 2002a; Keller, et al. 2002b), putamen (Keller, et al. 2011a), prefrontal region (Keller, et al. 2009a), planum temporale (Keller, et al. 2007; Keller, et al. 2011b), Broca’s area (Keller, et al. 2007; Keller, et al. 2009b) and insula (Keller, et al. 2011b). The CE is essentially a statistical estimate of how accurate the stereological volume estimation is for each structure. Separation between test points on the square grid used for point counting was 0.312 cm (i.e., 4 pixels) and slice interval was 1 mm (singular axial MR sections). Thalamic transect area was obtained by multiplying the total number of points recorded by the area corresponding to each test point. An estimate of thalamic volume was obtained as the sum of the estimated areas of the structure transects on consecutive systematic sections multiplied by the distance between sections. Between approximately 400–550 points were recorded on approximately 20 systematic random sections.

B. FreeSurfer

FreeSurfer software (http://surfer.nmr.mgh.harvard.edu/) was used to obtain thalamic volumes for all subjects using an observer-independent approach, which could be contrasted with the manual stereological measurements of the thalamus. Thalamic segmentations are based on the assignment of neuroanatomical labels to each voxel in an MR image based on the probabilistic information automatically estimated from a manually labelled training set. The methods of the automated volumetric approach have been described in detail previously (Fischl, et al. 2002), and the accuracy of automated labelling and volumetry of subcortical structures have been independently validated with respect to ‘gold standard’ manual volumetric techniques, predominantly for the hippocampus (Cherbuin, et al. 2009; Dewey, et al. 2010; Morey, et al. 2009; Pardoe, et al. 2009; Shen, et al. 2010; Tae, et al. 2008), and also of the amgydala (Dewey, et al. 2010; Morey, et al. 2009) and striatum (Dewey, et al. 2010). To our knowledge, there has been no independent comparison of the automated thalamic volumetry offered by FreeSurfer and a manual volumetric method (although thalamic tracings were compared with the performance of FreeSurfer in the original methods paper by Fischl et al. (2002)). Figure 1 shows the comparison of automated labelling of the thalamus (and extra-thalamic structures) in an individual control subject using FreeSurfer relative to stereological volume estimation of the thalamus in the same subject.

FreeSurfer analyses were performed on a Mac Pro (Version OS X 10.6.6, 32 GB, 2 × 2.93 GHz 6-Core Intel Xeon (HT)), which permitted the FreeSurfer ‘recon-all’ function (for cortical reconstruction and brain segmentation; http://surfer.nmr.mgh.harvard.edu/fswiki/recon-all) to complete 23 participants in less than 20 h. After the ‘recon-all’ function, the neuroanatomical labels were inspected for accuracy in all patients and controls. Despite that FreeSurfer permits manual editing to improve subcortical segmentation, no obvious errors in the automatic labelling were observed for any subject, and so all data obtained from FreeSurfer analyses were 100 % automated and not influenced by manual intervention.

### Statistical Analyses

Two-way mixed intra-class correlation coefficients for absolute agreement (Shrout and Fleiss 1979) were used to determine inter-rater agreement between two human raters using manual stereology and FreeSurfer for volume estimation of the thalamus in ten randomly selected controls using the statistics software SPSS (Version 18, www.spss.com). Intra-class correlations were subsequently performed between stereological volumes obtained by one human rater and FreeSurfer volumes for the entire sample of patients and controls (*n* = 72). Univariate ANOVAs were used to investigate patient-control differences in volumes, and corrected for multiple comparisons using Statistica version 9.1, (Stat Soft. Inc, www.statsoft.com).

## Results

A. Stereology vs FreeSurfer: Consistency between volumetric measures

**Fig. 2:**
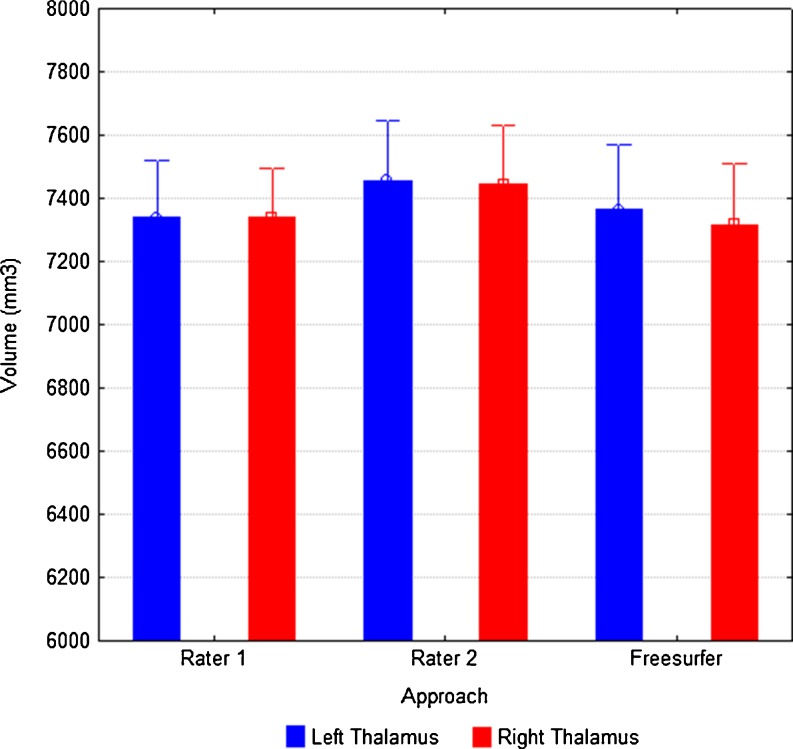
Inter-rater consistency in stereological volume estimation of the left (*blue*) and right (*red*) thalamus, and relation to volume estimates obtained from FreeSurfer. Stereological volume estimates are reproducible, and FreeSurfer is entirely consistent with the volumes obtained with both rater one and rater two. Error bars indicate the 95 % confidence intervals

Figure 2 shows the comparison of the three approaches (rater one (R1) for stereology, rater two (R2) for stereology and FreeSurfer) to estimate thalamic volume in the randomly selected ten subjects. This inter-rater / between-technique analysis indicates consistency across measures, and most notably, that FreeSurfer performed at least as consistent as R2 relative to R1. Mean (SD) left and right thalamic volume was 7339.6 mm^3^ (567.3) and 7339.2 mm^3^ (489.3) for R1, 7456.3 mm^3^ (597.3) and 7444.3 mm^3^ (590.6) for R2, and 7365.0 mm^3^ (641.3) and 7317.6 mm^3^ (610.0) for FreeSurfer, respectively. Intra-class correlations across raters and approaches are presented in Table 1. Measurements between R1 and R2, and between manual raters and FreeSurfer, achieve high intra-class correlations (all >0.9).

**Table 1:** Inter-rater intra-class coefficients for volumetric measures

	Rater 1	Rater 2	FS
Left thalamus			
Rater 1	−	0.977	0.969
Rater 2	0.977	−	0.925
FS	0.969	0.925	−

Right thalamus			
Rater 1	−	0.952	0.923
Rater 2	0.952	−	0.916
FS	0.923	0.916	−

**Fig. 3:**
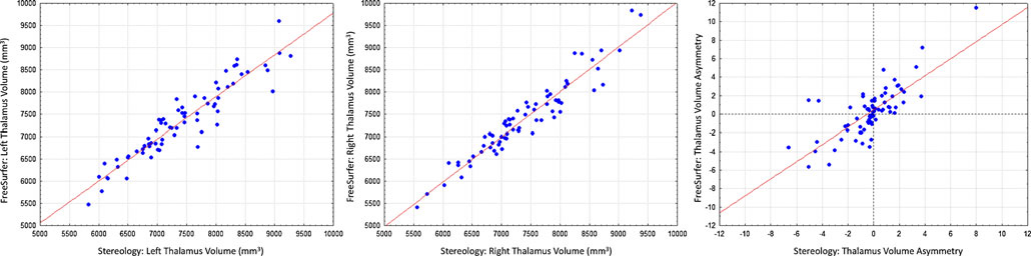
Relationship between stereological and FreeSurfer volume estimates of the left **a** and right **b** thalamus in the whole study sample. The relationship between left-right asymmetries determined for each individual participant by each technique is shown in **c**. Although stereology and FreeSurfer determined left-right asymmetries of the thalamus in the same direction for the vast majority of subjects (lower left and upper right quandrants), there were some disassociations (*top left quadrant*)

Figure 3 presents the relationship between stereological (R1) and FreeSurfer estimates of left and right thalamus volume across the entire sample of 72 subjects investigated in the present study. It is immediately obvious that the two methods yield consistent thalamic volumes. Intra-class correlations revealed a slightly reduced level of consistency between human and FreeSurfer analysis of the entire sample relative to the sub-sample of ten subjects (left thalamus = 0.812, right thalamus = 0.881). Mean (SD) left and right thalamic volume was 7422.4 mm^3^ (824.3) and 7390.1 mm^3^ (805.8) for stereology and 7343.6 mm^3^ (824.3) and 7388.3 mm^3^ (844.0) for FreeSurfer. There was no difference between the volume of the left and right thalamus across all 72 subjects using stereology (*F* = 0.09, *p* = 0.82) or FreeSurfer (*F* = 0.10, *p* = 0.75) (and no differences when patients and controls were separated, *p* > 0.80). Although there were occasional differences in the direction of inter-hemispheric thalamic volume asymmetry in individual cases between stereology and FreeSurfer (Fig. 3, right panel), this did not represent a statistically significant group effect (*F* = 1.46, *p* = 0.23).

B. Stereology vs Freesurfer: Identification of thalamic atrophy in JME

**Fig. 4:**
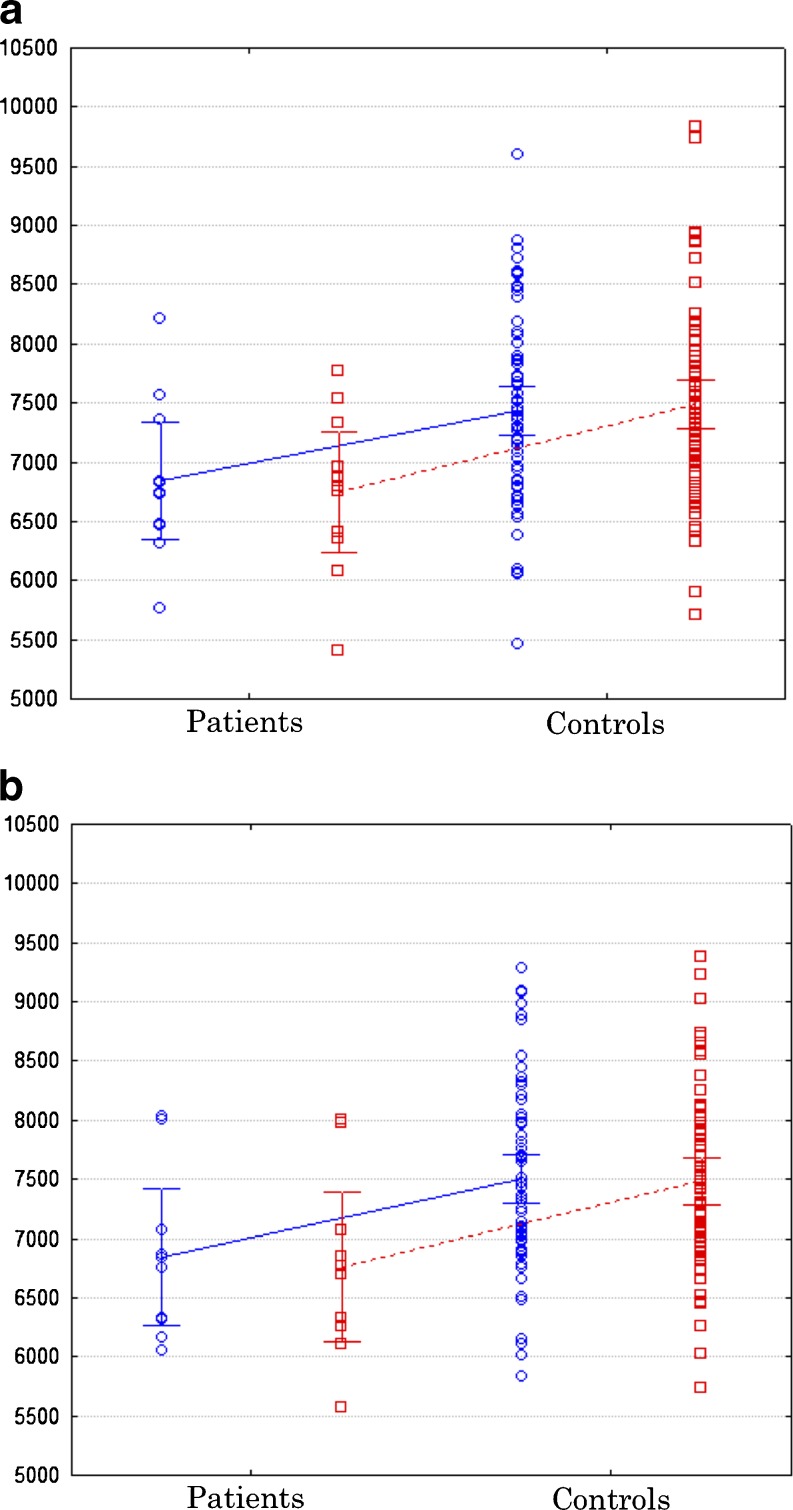
Volume reduction of the left (*blue circles*) and right (*red squares*) thalamus in patients with JME relative to healthy controls using stereology **a** and FreeSurfer **b**. Error bars indicate the 95 % confidence intervals

Both techniques were equally sensitive in detecting bilateral thalamic volume atrophy in patients with JME relative to controls (Fig. 4). Using stereology, mean (SD) left and right thalamic volume was 6843.2 mm^3^ (746.6) and 6763.3 mm^3^ (824.0) in patients with JME, and 7507.8 mm^3^ (805.6) and 7482.6 mm^3^ (767.2) in controls, respectively. Volume reduction in patients was found to be statistically significant for the left (F(1,70) = 5.43, *p* = 0.02) and right (F(1,70) = 6.77, *p* = 0.01) thalamus compared to controls. Using FreeSurfer, mean (SD) left and right thalamic volume was 6803.4 mm^3^ (732.8) and 6750.0 mm^3^ (714.3) in patients with JME, and 7430.7 mm^3^ (809.9) and 7491.3 mm^3^ (822.3) in controls, respectively. FreeSurfer thalamic volumes were similarly smaller in patients relative to controls in the left (F(1,70) = 4.76, *p* = 0.03) and right (F(1,70) = 7.36, *p* = 0.008) hemispheres.

## Discussion

The volume of the thalamus is a notoriously difficult metric to estimate reliably given the low contrast between thalamic gray matter and adjacent white matter on T1-weighted MR images, which is a particular challenge for automated MR image analysis methods (Amini, et al. 2004). Only by comparing such automated methods with manual investigator-intensive methods can we establish the reliability of volume estimates. The present study provides important data indicating the specificity and validity of automated thalamic volume estimation using FreeSurfer software. In particular, further to demonstrating consistency between stereological and FreeSurfer volume estimates of the thalamus in healthy subjects and neurological patients, we demonstrate that the extent of agreeability between stereology and FreeSurfer is equal to the agreeability between two human anatomists estimating thalamic volume using stereological methods.

FreeSurfer software is now a frequently used tool for the estimation of subcortical structure volume. At the time of writing, a pubmed search using “Freesurfer” and “volume” yields 87 articles (October 2011). The vast majority of these articles are application studies, particularly in neurological disorders, and only a few have sought to evaluate the validity of volume measurements. Various levels of consistency between FreeSurfer and manual ROI methods have been reported for the hippocampus (Cherbuin, et al. 2009; Dewey, et al. 2010; Morey, et al. 2009; Pardoe, et al. 2009; Shen, et al. 2010; Tae, et al. 2008), amgydala (Dewey, et al. 2010; Morey, et al. 2009) and striatum (Dewey, et al. 2010). Dewey et al. (2010) performed a series of comparisons between the fully automated techniques of FreeSurfer and Individual Brain Atlases using Statistical Parametric Mapping (IBASPM) with auto-assisted manual tracings of the hippocampus, amygdala, putamen and caudate. The authors report that FreeSurfer segmentations exhibited significantly higher mean spatial overlap with auto-assisted tracings in all structures compared to IBASPM using dice coefficients. We were not in a position to perform spatial overlap analyses of the thalamus given that stereology and FreeSurfer are two inherently distinct MR image analysis approaches. However, this is one of the primary strengths of the data presented here, insomuch that a reliable volume estimate obtained using a gold-standard (non-voxel labelling) manual approach on MR images without automated spatial transformations (i.e. in native space) is comparable to a fully automated approach that requires spatial transformations in order to label an ROI and obtain a volume. Our interest was with respect to the reliability of the volume estimate of the thalamus.

To our knowledge, the present study is the first to independently provide data validating the application of FreeSurfer to obtain automated volumes of the left and right thalamus. Based on the congruence between the data obtained from FreeSurfer and manual stereology—the latter of which is considered to represent the ‘gold standard’ approach due to the requirement of an expert anatomist—we recommend the use of FreeSurfer software for accurate volumetric quantification of the thalamus using high-resolution T1-weighted MRI. The removal of an expert anatomist for volumetric analyses is cost effective and time efficient, particularly in large-scale volumetric studies. Importantly, we demonstrate that the automated technique is as sensitive in detecting pathological alterations of the thalamus relative to stereology, which promotes the use of FreeSurfer in neurological contexts.

There are two additional issues that should be highlighted. Measurements made in the present study were of global thalamic volume. The thalamus is composed of lamellae that segregate multiple nuclei with distinct connections and functions, which are likely to be differentially affected in various neurological and neuropsychiatric disorders. For example, in disorders where the thalamus is implicated in patients also exhibiting deficits in frontal lobe functioning—such as in JME (Pulsipher, et al. 2009)—it would be expected that anterior thalamic nuclei that project to the frontal lobe would be preferentially affected (Deppe, et al. 2008). In such circumstances it will be interesting to investigate structural alterations of differential thalamic subregions, which are measures that the techniques applied in the present study cannot provide. There are other techniques that may provide the basis for quantitative measurements of thalamic subregions based on DTI and quantitative T1 and T2 imaging (Behrens, et al. 2003; Johansen-Berg, et al. 2005; Traynor, et al. 2011). Secondly, the global estimates of thalamic volume using FreeSurfer in the present study was obtained on a Philips Intera T30 3 T MRI system, requiring no additional manual edits for (obvious) incorrect labelling of the thalamic ROI after the application of our in-house image inhomogeneity and resampling algorithm. Different MRI systems and head coils may have different image contrast characteristics that can potentially affect the performance of automated MR image analysis techniques. However, reproducibility of FreeSurfer estimated thalamic volume from serially acquired MR images on the same MR system is high (Jovicich, et al. 2009; Morey, et al. 2010), and MR system manufacturer has been shown to have little effect on volume estimates (Jovicich, et al. 2009).

In summary, this study provides convincing evidence for the reliability of global thalamic measurements using FreeSurfer in healthy and damaged thalami. The use of this software is cost effective and particularly advantageous in large-scale cross-sectional studies and longitudinal investigations in neurological settings.

## Information Sharing Statement

FreeSurfer software is publicly and freely available from the FreeSurferWiki resource (http://surfer.nmr.mgh.harvard.edu/fswiki/FreeSurferWiki), which is developed and maintained at the Martinos Center for Biomedical Imaging (http://www.nmr.mgh.harvard.edu/martinos/noFlashHome.php). All software, information and support are provided online at the FreeSurferWiki webpage. Easymeasure software for volume estimation using stereology is freely available from the authors of this manuscript upon request. Dr. Mike Puddephat developed Easymeasure software at the University of Liverpool, UK. Further information can be found at (http://www.easymeasure.co.uk/).
